# Frailty and its association with health-related quality of life among older cancer patients: an evidence-based study from China

**DOI:** 10.1186/s12955-022-02032-7

**Published:** 2022-08-19

**Authors:** Mingzhu Su, Nengliang Yao, Meimei Shang, Yuzhen Shen, Tingting Qin, Jialin Wang, Xiaojie Sun

**Affiliations:** 1 Centre for Health Management and Policy Research, School of Public Health, Cheeloo College of Medicine, Shandong University, Jinan, 250012 Shandong China; 2 NHC Key Lab of Health Economics and Policy Research, Shandong University, Jinan, 250012 Shandong China; 3 Shandong Cancer Hospital and Institute, Shandong First Medical University and Shandong Academy of Medical Sciences, Jinan, 250117 Shandong China; 4 Taian City Center Hospital, Taian, 271099 Shangdong China; 5 School of Public Health, Capital Medical University, Beijing, 100069 China

**Keywords:** Cancer, Elderly, Frailty, Health-related quality of life

## Abstract

**Background:**

There is limited information about the population characteristics and adverse health outcomes of older cancer patients in China. This study aimed to describe the prevalence of frailty and examine the association between frailty and health-related quality of life (HRQoL) among older cancer patients.

**Methods:**

This was a cross-sectional study involving older patients diagnosed with cancer in two tertiary hospitals in Shandong Province, China. Frailty was assessed using Geriatric 8 (G-8). HRQoL was measured using the five-level EuroQol-5-dimension (EQ-5D-5L) questionnaire. The Tobit regression model and logistic regression model was used to identify the relationship between frailty and HRQoL.

**Results:**

Of the 229 older patients, 175 (76.4%) were frail. Frail patients had lower EQ-5D-5L utility scores than those who were non-frail (0.830 vs. 0.889; *P* = 0.004). After adjustments for sociodemographic and cancer-related variables, frailty was statistically associated with worse health-related quality of life (*OR* = 6.024; *P* = 0.001).

**Conclusion:**

Frailty was associated with deteriorated HRQoL in older patients with cancer. Early frailty screening and preventive interventions are essential for improving quality of life through decision-making or pretreatment optimization in geriatric oncology.

## Plain English summary

The majority of cancer cases and cancer mortality occur in older adults. Aging is a heterogenic process, which results in great diversity in older cancer patients concerning physical, psychological, and social status. There is limited information about the population characteristics and adverse health outcomes of older cancer patients in China, and the magnitude of this problem is unclear, creating challenges in understanding health disparities in geriatric cancer care. In this study, we have described the prevalence of frailty and explored the relationship between frailty and health-related quality of life (HRQoL). This study demonstrated that frailty, not being currently married, advanced cancer stage, and cancer site were significant factors influencing the lower HRQoL. Furthermore, frail individuals had a higher probability belonging to the lowest HRQoL quartile group. This study is considered as the first step in highlighting the importance of staging the aging and assessing quality of life among older cancer patients in China.

## Introduction

Global cancer statistics indicated that there would be an estimated 19.3 million new cancer cases and 9.9 million deaths from cancer all over the world in 2020; and approximately 23.7% of all cancer cases and 30.1% of all cancer deaths cases occurred in China [1]. Lung, colorectal, stomach, breast, and liver cancer were the most common cancers in China, which contributed to the major burden of cancer [2, 3]. Due to the aging of the population and changes in disease patterns, nearly 60% of cancer cases and 70% of cancer mortality occurred in patients aged 60 years and over in China [2]. Considering the unprecedented rate of population aging and the increasing incidence of cancer, the number of older adults diagnosed with cancer will continually increase in the future.

Aging is a heterogenic process, which results in great diversity in older adults concerning physical, psychological, and social status [4–6]. Frailty can represent the individual’s biological age rather than chronological age, and is defined as “a state of cumulative decline in multiple physiological systems, resulting in erosion of homeostatic reserve and increased vulnerability to poor health status following a stress” [7]. Though most oncologists have little or no training in geriatric medicine, they often encounter common age-related diseases and health problems, which leads to more challenging medical decision-making in the geriatric oncology continuum [8]. Cancer, its treatment, and the lasting effects of that treatment are important stressors affecting declines in functional reserves across physiological systems [9]. Thus, the concept of frailty is particularly important for older cancer patients. Previous studies have already shown that frailty was associated with a high risk of the occurrence of complications, prolonged hospital stays, and worse overall survival rate in older adults with cancer [10–12]. These outcome indicators are also clinically relevant. Collecting and considering patients’ reported outcomes during medical decision-making can affect patients’ health-related quality of life (HRQoL), as well as their experiences of symptoms, functioning, and values[13]. In this study, the definition of HRQOL is the value assigned to duration of life as modified by the impairment, functional states, perception and social opportunities that are influenced by disease, injury, treatment, or policy[14].

Well-validated frailty screening instruments have been used in oncology care in developed countries to measure the impact of age-related concerns on functioning and well-being. Previous research demonstrated that frailty was associated with lower HRQoL in oncological cohorts, and patients reported that HRQoL was increasingly considered a significant outcome measure for cancer treatment [15–17]. In China, only a few studies have evaluated the relationship between frailty in older adults with cancer and their HRQoL. Prior studies on frailty among cancer patients in hospitals have focused almost exclusively on the acute treatment toxicity rather than examining patient-reported health status [18]. Most studies have focused on a single cancer type in clinical trials and few studies have focused on frailty screening instruments in geriatrics oncology care [19, 20]. There is limited information about the population characteristics and adverse health outcomes of older cancer patients in China, and the magnitude of this problem is unclear, creating challenges in understanding health disparities in geriatric cancer care. Therefore, the purpose of the study was to describe the prevalence of frailty in Chinese older patients with cancer and to analyze the association between frailty and HRQoL.

## Methods

### Data source

We used convenience sampling in a general tertiary hospital and a cancer tertiary hospital in Shandong Province, China from July to September 2020. To improve the response rates, the nursing and public health postgraduates were hired to conduct this hospital survey and all them received a formal intensive training before the in-person interviews. Inclusion criteria of participants in this study were as follows: (a) previously diagnosed with the most common cancers in China (such as lung, stomach, colorectal, esophageal cancers); (b) 60 years or older at the time of diagnosis; (c) cancer treatment ongoing (at least three months post-diagnosis); (d) ability to give written informed consent. Potential participants were excluded if they had cognitive deficits, mental illness, or other problems that would hinder their participation in the questionnaire survey. Participants were interviewed face-to-face by trained investigators at their wards. Ethical approval for this study was obtained from the Ethics Committee of the Centre for Health Management and Policy Research, at Shandong University (ECSHCMSDU20200101).

### Frailty

The G-8, a geriatric screening tool, is a simple and useful instrument for identifying geriatric risk profiles in older cancer patients [21]. We assessed frailty using the modified G-8 screening tool (ranges from 0 to 17) with a frailty cutoff of ≤ 14 [22]. Patients with a G-8 score lower than or equal to 14 were considered to be frail. This tool has shown good sensitivity for geriatric impairments across multiple domains (food intake, weight loss, mobility, Body Mass Index (BMI), medication use, self-reported health status, 4-Item IADL (Instrumental Activity of Daily Living), and age) [22]. Frailty in older adults was treated as dichotomous independent measures (not frail vs frail).

### Health-related quality of life

HRQoL was measured using the five-level EuroQol-5-dimension (EQ-5D-5L) questionnaire. Although there are various validated instruments for assessing HRQoL, the EQ-5D has demonstrated high internal validity and high test–retest reliability [23]. EQ-5D-5L is a newer version of the EQ-5D, a brief, generic, preference-based instrument developed by the Euro-Qol Group [24]. The first part comprised five dimensions: (a) mobility; (b) self-care; (c) usual activities; (d) pain/discomfort; (e) anxiety/depression, each with five levels corresponding to “no(t)/ slight/ moderate/severe/unable or extreme,” resulting in a total of 5^5^ (3125) unique composite health states [24]. The Chinese utility values for 11,111 (the best health state) and 55,555 (the worst health state) are 1 and − 0.391, respectively [25]. The second part was the EQ-visual analog scale (EQ-VAS), in which the participants described their health status using a VAS scale from 0 (worst health status) to 100 (best health status). The main dependent variable in our study was health utility score, which was treated as a continuous independent measure.

### Other variables

Cancer site and stage were obtained from the electronic medical records. Chronic diseases such as self-reported histories of heart disease, pulmonary disease, hypertension, diabetes, chronic lung disease, and neurological diseases were also assessed from the survey data. Additionally, the survey included questions on socioeconomic status and disease-related characteristics (age, sex, marital status, level of education, health insurance, monthly household income, treatment modalities, time of diagnosis).

### Statistical analysis

Mean and standard deviations (SD) were calculated for continuous variables; and frequencies and percentages were calculated for categorical variables. Due to a skewed distribution, the Mann–Whitney U test and the Kruskal–Wallis test were conducted to test the differences in EQ-5D-5L utility scores and VAS-scores among various subgroups. The Tobit model, also called a censored regression model, is designed to estimate linear relationships between variables when there is either left- or right- censoring in the dependent variable [26]. Due to a number of the utility scores clustered at the limiting value (e.g., 11,111 indicates the best health state), the Tobit regression model was employed to examine the influence of socio-demographic variables, clinical characteristics, and frailty status on the utility scores of cancer patients. We also conducted logistic regression analyses for the odds of scoring in the bottom 25% of the EQ-5D-5L utility scores [27]. All regression analyses included clinically important socioeconomic variables and cancer variables, which were used to control for confounding covariates. All statistical analysis were performed using the R software (version 3.3.1). All statistical tests in this study were two‐sided (*P* < 0.05).

## Results

About 260 eligibility cancer cases were selected from electronic medical record. Among non-participants (N = 26), refusal to participate was the main reason, with other reasons including sleep and absence from the hospital room. Among 234 participants, 5 respondents (2%) did not complete the questionnaire; and 229 respondents (98%) who completed the questionnaire were included in the final analysis. Table 1 below summarizes the characteristics of the participants. The mean age of the participants was 68 years (SD: 6.4; range: 60–117). In total, 79.5% of participants were men, 93.9% were married, and most were uneducated or had a primary school education (46.3%). Monthly household income was less than 1000 Chinese Yuan for 20.1% (N = 46) of participants, while 17.9% reported an income level of more than 5000 Chinese Yuan. The time interval between cancer diagnosis and survey date was 12 months on average. Comorbidities were present for 34.5% of the participants. Of the sample, 45.0% had lung cancer, 19.6% had stomach cancer, and 12.2% had esophageal cancer. Participants with an advanced cancer stage (stage III-IV) at diagnosis were 62.5%. Treatments received by participants included surgery (51.5%), chemotherapy (67.2%) and radiation therapy (24%). Time from diagnosis to survey was 12.2 months on average (SD: 12.5; range: 3–80).

**Table 1:** Characteristics of the participants (N = 229)

Characteristics	N	%
*Age group*		
60–64	74	32.3
65–69	79	34.5
70–74	46	20.1
≥ 75	30	13.1
*Sex*		
Male	182	79.5
Female	47	20.5
*Marital status*		
Married	215	93.9
Not married	14	6.1
*Education level*		
Uneducated and primary school	106	46.3
Middle school	67	29.3
High school and above	56	24.4
*Health Insurance*		
UEBMI	77	32.6
URRBMI	149	65.1
Other	3	1.3
*Monthly household income, Chinese Yuan*		
< 1000	46	20.1
1000–3000	72	31.4
3000–5000	70	30.6
5000–10,000	41	17.9
*Cancer site*		
Lung	103	45.0
Stomach	45	19.6
Esophagus	28	12.2
Colorectal	24	10.5
Other (Liver, head and neck, bladder)	29	12.7
*Cancer stage now*		
I	48	20.9
II	38	16.6
III	62	27.1
IV	81	35.4
Type of treatment^*^		
Surgery	118	51.5
Chemotherapy	154	67.2
Radiation	55	24.0
*Comorbidity*		
0	150	65.5
1	58	25.3
≥ 2	21	9.2
*Diagnosis year*		
≤ 1 year	154	67.2
≥ 2 years	75	32.8

^*^Categories are not mutually exclusive because most patients received a combination of treatments

Abbreviations: *UEBMI* Urban Employees' Basic Medical Insurance, *URRBMI* Urban–Rural Residents' Basic Medical Insurance

The mean G-8 total score was 12.4 (SD: 2.5; range: 6–17). And 76.4% participants (G-8 score ≤ 14) were classified as frail. The prevalence of each frail component was as follows: problems with nutritional status 55.9%, self-reported health status 46.3%, medication use 14.4%, mobility 7.9%, and age 3.0%. Detailed information is presented in Table 2.

**Table 2:** Frequency of item response in G-8 (N = 229)

Items	The proportion with problems (%)
Severe or moderate decrease in food intake over the past 3 months	55.9
Weight loss > 3 kg during the last 3 months	28.4
Mobility (can’t goes out)	7.9
Body Mass Index < 19	15.7
Takes more than 3 medications per day	14.4
Compared to other people of the same age, self-reported health not as good or does not know	46.3
4-Item IADL (dependent)	
Use phone	4.4
Use mode of transportation	19.2
Have responsibility for medication	3.5
Handle finances	19.2
Age > 80 years old	3.0

The mean EQ-5D-5L utility score was 0.835 (SD: 0.165; range: − 0.007–1), and the mean EQ-VAS score was 76.1 (SD: 14.30; range: 20–100). The percentages of utility scores for 1 (right censored response) and − 0.391 (left censored response) were 22.3% and 0%, respectively. Fifty-one participants (22.3%) reported no problems on any of the five dimensions. The proportion of participants reporting pain/discomfort problems was highest (65.9%), followed by anxiety/depression (64.2%). Only 11.8% of participants, reported problems with self-care.

Table 3 shows the mean scores from the EQ-5D measures categorized for various subgroups and frailty statuses. In the bivariate analysis, the difference in the EQ-5D utility scores was not statistically significant among different socio-demographic subgroups (such as gender, educational level, and more). The difference in EQ-5D utility scores was statistically significant among the subgroups with different cancer sites or stages (*P* < 0.001). Those who had chemotherapy and radiation treatment, had a lower utility score than those who did not (*P* = 0.001; *P* = 0.039). As for comorbidity, those with existing chronic diseases (*P* = 0.037) had a lower utility score than those without them. Frail participants had lower EQ-5D-5L utility scores (0.830 ± 0.174) than the not frail group(*P* = 0.018). There were no significant differences in VAS-scores by socio-demographic and cancer-related characteristics. Particularly, frail patients (75.29 ± 14.15) were not statistically significant in VAS-score (*P* = 0.118).

**Table 3:** The comparisons of EQ-5D-5L utility scores by social demographic, clinical and frailty subgroups (N = 229)

Background characteristics	Mean	SD	Z/χ^2^	P
Age group (Years)			3.338	0.338
60–64	0.859	0.149		
65–69	0.844	0.188		
70–74	0.848	0.107		
≥ 75	0.798	0.209		
Sex			− 0.552	0.581
Male	0.847	0.156		
Female	0.828	0.197		
Marital status			− 1.497	0.134
Married	0.840	0.169		
Not married	0.905	0.086		
Education level			4.518	0.104
Uneducated and primary school	0.818	0.183		
Middle school	0.878	0.125		
High school and above	0.851	0.166		
Health Insurance			− 1.351	0.177
UEBMI	0.858	0.166		
URRBMI	0.836	0.167		
Monthly household income, Chinese Yuan			0.865	0.834
< 1000	0.820	0.177		
1000–3000	0.858	0.142		
3000–5000	0.843	0.176		
5000–10,000	0.844	0.174		
Cancer site			36.331	< 0.001
Lung	0.800	0.162		
Stomach	0.913	0.109		
Esophagus	0.790	0.262		
Colorectal	0.932	0.085		
Other (Liver, head and neck, bladder)	0.870	0.109		
Cancer stage			24.136	< 0.001
I	0.910	0.098		
II	0.868	0.212		
III	0.783	0.196		
IV	0.839	0.129		
Type of treatment				
Surgery	0.866	0.136	− 1.894	0.058
	0.819	0.190		
Chemotherapy	0.829	0.153	− 3.434	0.001
	0.873	0.186		
Radiation	0.803	0.201	− 2.061	0.039
	0.856	0.151		
Comorbidity			6.578	0.037
0	0.835	0.177		
1	0.881	0.123		
≥ 2	0.797	0.165		
Diagnosis year			− 1.700	0.089
≤ 1 year	0.851	0.167		
≥ 2 years	0.828	0.163		
Frailty status			− 2.366	0.018
Not frail	0.889	0.124		
Frail	0.830	0.174		

Abbreviations: *UEBMI* Urban Employees' Basic Medical Insurance, *URRBMI* Urban–Rural Residents' Basic Medical Insurance

Table 4 shows the results from the multinomial Tobit regression, analyzing the association between frailty and EQ-5D-5L utility scores. The variation inflation factor (VIF) ranged from 1.102 to 2.240, and the tolerance ranged from 0.446 to 0.907, indicating no multicollinearity. Lower EQ-5D-5L utility scores were associated with frail status (*P* = 0.004), a more advanced cancer stage (III, IV) at diagnosis (*P* < 0.001), stomach cancer (*P* < 0.001) and colorectal cancer (*P* < 0.001), while being married (*P* = 0.018) was statistically associated with higher utility scores. Table 4 also presents the logistic regression results, estimating the probabilities belonging to the lowest quartile group in terms of EQ-5D-5L utility scores. In this logistic regression model, VIF ranged from 1.080 to 2.493, and the tolerance ranged from 0.401 to 0.925, indicating no multicollinearity. After adjustments for socio-demographic and cancer-related factors (sex, marital status, monthly household income, educational level, health insurance, cancer site, cancer stage, time since diagnosis, and comorbidity), older cancer patients who were frail had a significantly higher probability belonging to the lowest quartile group (*OR* = 6.024; *95% CI* 2.194–19.429; *P* = 0.001).

**Table 4:** Associations between Frailty and Health-related Quality of Life among older cancer patients: Tobit regression and Logistic regression (N = 229)

	EQ-5D-5L utility scores				EQ-5D-5L lower quartiles			
Variables	Estimate	SE	t	P	Estimate	OR	95%CI	P
*Sex*								
Male	Ref				Ref			
Female	− 0.003	0.033	− 0.086	0.931	− 0.094	0.910	0.348–2.291	0.844
*Marital status*								
Married	Ref				Ref			
Not married	0.127	0.053	2.376	0.018	− 0.922	0.398	0.056–1.785	0.275
*Education level*								
Uneducated and primary school	Ref				Ref			
Middle school	0.055	0.031	1.761	0.078	− 0.784	0.456	0.173–1.146	0.102
High school and above	0.022	0.036	0.612	0.541	0.110	1.116	0.397–3.110	0.832
*Health insurance*								
UEBMI	Ref				Ref			
URRBMI	− 0.029	0.034	− 0.862	0.388	0.159	1.172	0.430–3.244	0.756
*Monthly household income, Chinese yuan*								
< 1000	Ref				Ref			
1000–3000	0.020	0.036	0.550	0.582	− 0.723	0.485	0.180–1.284	0.147
3000–5000	− 0.037	0.040	− 0.926	0.354	− 0.232	0.793	0.263–2.371	0.678
5000–10,000	0.002	0.050	0.046	0.963	− 0.855	0.425	0.102–1.682	0.229
*Cancer site*								
Lung	Ref				Ref			
Stomach	0.143	0.035	4.081	0.000	− 1.687	0.185	0.048–0.559	0.006
Esophagus	0.011	0.040	0.268	0.789	− 0.552	0.576	0.182–1.670	0.323
Colorectal	0.167	0.045	3.738	0.000	− 0.827	0.437	0.131–1.318	0.156
Other	0.096	0.041	2.307	0.021	− 1.455	0.233	0.047–0.861	0.044
*Cancer stage*								
I	Ref				Ref			
II	− 0.071	0.042	− 1.692	0.091	0.178	1.187	0.275–5.106	0.814
III	− 0.150	0.038	− 3.885	0.000	1.369	3.930	1.270–14.158	0.024
IV	− 0.131	0.038	− 3.482	0.000	1.250	3.859	1.214–14.239	0.029
*Comorbidity*								
0	Ref				Ref			
1	0.042	0.029	1.440	0.150	− 0.178	0.837	0.342–1.952	0.687
≥ 2	− 0.034	0.042	-0.803	0.422	0.343	1.409	0.438–4.455	0.558
*Diagnosis year*								
≤ 1	Ref				Ref			
≥ 2	0.000	0.028	− 0.004	0.997	0.458	1.580	0.735–3.413	0.240
*Frailty status*								
Not Frail	Ref				Ref			
Frail	− 0.088	0.031	− 2.861	0.004	1.796	6.024	2.194–19.429	0.001

Abbreviations: *UEBMI* Urban Employees' Basic Medical Insurance; *URRBMI* Urban–Rural Residents' Basic Medical Insurance

## Discussion

The purpose of this study was to examine the association between frailty and HRQoL in older Chinese patients with cancer. Our findings showed that frail cancer patients had lower EQ-5D-5L utility scores than non-frail patients, demonstrating that frailty, not being currently married, advanced cancer stage, and cancer site were significant factors influencing the HRQoL. Furthermore, compared to non-frail participants, frail individuals had a higher probability belonging to the lowest HRQoL quartile group. This study is considered as the first step in highlighting the importance of staging the aging and assessing quality of life among older cancer patients using geriatric screening tools and generic preference-based instruments in China.

The valid health utility value from general populations takes into account Chinese preferences and perceptions of different diseases, and provides an available standard means to ensure comparability between outcomes from other diseases [25]. Healthy utility values stratified by frailty status can help us to understand and explain the quality of life for older cancer patients and also provide essential baseline data for cancer-treatment-related economic evaluations. Our data showed that a high proportion of older cancer patients had pain/discomfort problems, and frailty status negatively influenced HRQoL. The mean utility score for the frail group was 0.830, which was lower than the EQ-5D-5L norms for the Chinese older population (0.940) [28]. Oncologists and geriatricians can use this finding for medical decision-making or risk stratification for cancer treatment and care, taking patient-reported health status into account. And more attention should be focused on early frailty screening, as preventive strategies and interventions are necessary to improve quality of life in clinical routine care.

Frailty has gradually emerged as a significant health evaluation indicator during the medical decision-making for elderly cancer patients at oncology departments in developed countries [29]. Screening tools assessing frailty status usually incorporated age, geriatric syndromes, nutritional status, and mobility. The prevalence of frailty in cancer patients in this study was 76.5%, which is similar to a study on frailty in Belgium that included 170 older cancer patients, but is higher than a study by Soubeyran and colleagues (68.4%), and both of them used the G-8 [30, 31]. The difference in frailty prevalence estimations may be linked to the variations in study design or sample characteristics. The G-8 is strongly associated with treatment complications and survival in cancer samples; however, its relationship with HRQoL has rarely been explored. Important health and disease outcomes for older adults with a diagnosis of cancer include not only tumor shrinkage and progression-free survival, but also the impact of treatment on geriatric domains and HRQoL. Several studies have indicated that frail individuals with cancer had an increased risk for decline in HRQoL [32, 33]. Generally speaking, quality of life decreases with age; however, in this study, we could not assess age-adjusted analysis to examine its association with EQ-5D-5L utility scores. Because age is a domain with the G-8 scoring systems. Although this study provided new evidence for the strong relationship between frailty and lower HRQoL in China, this association needs to be further explored more comprehensively in the future.

This study also indicated that advanced cancer stage, as well as frailty, were associated with lower quality of life, which has been suggested by other many studies [34, 35]. In general, older patients were less likely to trade increased survival duration for reductions in HRQoL in medical decision-making [36]. Unlike cancer stage, frailty can be altered by effective interventions to improve advanced-stage patients’ functionality and cancer prognosis. There were inconsistent results in our study with regard to the association between frailty and gender [37, 38]. Our findings showed that there was no difference in frailty between males and females, while females were more likely to be frail in other studies. The different findings in our study were possibly linked to the relatively small proportion (21%) of female older patients. Of the estimated 2.6 million new cancer cases in people aged 60 years and over, nearly 38% of all cancer occurs in older females in China [2]. Thus, our subgroup analysis by gender group may be underpowered to detect a statistically significant association.

The G-8 is a physically oriented screening tool with more than half of the items being related to nutrition status, weight loss, and comorbidities [16, 22]. A nutrition intervention may be needed to improve quality of life in cancer patients, based on our finding of a high prevalence of worse nutritional status, the main frail component in the G-8. Given that cancer is often a long-term and age-related illness, staging the aging in cancer patients should be considered as important as staging the cancer stage. As a large proportion of older cancer patients’ experiences frailty status that negatively impacts their quality of life, early frailty screening and preventive strategies are necessary to improve quality of life through decision-making and pretreatment optimization in the growing geriatric oncology population. Early frailty screening can allow oncologists to discriminate robust individuals from frailty individuals from the heterogeneous elderly patient population. Robust patients may benefit from standard treatment without extensive evaluations, and efforts can be focused on frailty patients who need careful medical attention to improve cancer care[31]. If medical resources are available, management of frailty patients should be multidisciplinary. If not, they should be offered at least cautious medical attention to improve their quality of life.

### Limitation

This study had several limitations. A limitation of this study is that the cancer sample was drawn from two tertiary hospital from Shandong Province, thus our participants may be under-represented. We assessed patients who were receiving treatment for at least three months post-diagnosis. Therefore, we were unable to conclude that newly diagnosed patients would provide similar results. The cancer sample in this study was heterogeneous in that it likely included patients at each cancer stage, from ongoing active treatment to those receiving palliative treatment for metastatic cancer or recurrences. Similarly, the differences with regard to the drivers of HRQOL among older patients with different cancer types should be examined because these drivers may vary by the type of disease or by the type of treatment. These limitations should be considered from the perspective that the goal of this study was to explore the association between frailty and HRQoL and not to identify all the populations at risk or the drivers of HRQoL. Secondly, this study was cross-sectional, so we could not determine if frailty status directly caused the lower quality of life in older Chinese patients. However, the strong findings demonstrating a relationship between frailty and HRQoL provide baseline data and insights for future studies. Thirdly, we used specific instruments to measure frailty, which can provide more sensitive results and enable evaluation of a specific condition; however, this tool restricted comparisons from being made with the general older population. The measurements of HRQoL and frailty screening tool present a certain amount of overlap for older adults. One possibility is that what determines poor health also determines frailty. Future research needs to further demonstrate the overlap between HRQoL and frailty in older age populations.

## Conclusion

In this study, we found that frailty was associated with an increased likelihood of having a utility score in the lowest quartile, compared to those without frailty. While our research this study provided new evidence for the strong relationship between frailty and lower HRQoL in China, needs to be further explored through future longitudinal and prospective studies.

## Data Availability

The data used in the present study from the authors upon reasonable request.

## Abbreviations

HRQoL: Health-related quality of life
G-8: Geriatric 8
EQ-5D-5L: The five-level EuroQol-5-dimension questionnaire
BMI: Body mass index
IADL: Instrumental activity of daily living
VAS: Visual analog scale
SD: Standard deviations
VIF: Variation inflation factor
